# How much of the invader's genetic variability can slip between our fingers? A case study of secondary dispersal of *Poa annua* on King George Island (Antarctica)

**DOI:** 10.1002/ece3.3675

**Published:** 2017-12-02

**Authors:** Maciej Wódkiewicz, Katarzyna J. Chwedorzewska, Piotr T. Bednarek, Anna Znój, Piotr Androsiuk, Halina Galera

**Affiliations:** ^1^ Biological and Chemical Research Centre Faculty of Biology University of Warsaw Warsaw Poland; ^2^ Institute of Biochemistry and Biophysics Polish Academy of Sciences Warsaw Poland; ^3^ Plant Breeding and Acclimatization Institute – National Research Institute Błonie Poland; ^4^ Department of Plant Physiology, Genetics and Biotechnology University of Warmia and Mazury in Olsztyn Olsztyn Poland

**Keywords:** alien species, amplified fragment length polymorphism, biological invasion, demographic processes

## Abstract

We studied an invasion of *Poa annua* on King George Island (Maritime Antarctic). The remoteness of this location, its geographic isolation, and its limited human traffic provided an opportunity to trace the history of an invasion of the species. *Poa annua* was recorded for the first time at H. Arctowski Polish Antarctic Station in the austral summer of 1985/6. In 2008/9, the species was observed in a new locality at the Ecology Glacier Forefield (1.5 km from “Arctowski”). We used AFLP to analyze the genetic differences among three populations of *P. annua*: the two mentioned above (*Station* and *Forefield*) and the putative origin of the introduction, *Warsaw* (Poland). There was 38% genetic variance among the populations. Pairwise Ф_PT_ was 0.498 between the *Forefield* and *Warsaw* populations and 0.283 between *Warsaw* and *Station*. There were 15 unique bands in the *Warsaw* population (frequency from 6% to 100%) and one in the *Station/Forefield* populations (which appears in all analyzed individuals from both populations). The Δ(*K*) parameter indicated two groups of samples: *Warsaw/Station* and *Forefield*. As indicated by Fu's *F*s statistics and an analysis of mismatch distribution, the *Forefield* population underwent a bottleneck and/or founder effect. The *Forefield* population was likely introduced by secondary dispersal from the *Station* population.

## 1 INTRODUCTION

Biological invasions present interesting evolutionary problems because they are stochastic events often involving small populations that can survive rapid habitat transitions (Colautti, Alexander, Dlugosch, Keller, & Sultan, 2017; Ellstrand & Schierenbeck, 2000; Lee, 2002). New ecological conditions encountered by individuals introduced into the new habitat may differ considerably from the conditions in their primary range. Therefore, natural selection and adaptation may be the key determinants of the success of invasion at the population level (reviewed in Facon et al., 2006; Schierenbeck & Ainouche, 2006). Substantial genetic variability is expected to favor adaptation in remote territories (Facon et al., 2006; Lavergne & Molofsky, 2007; Lee, 2002; Roman & Darling, 2007), while the rapid adaptation of invaders is common and generally not limited by genetic variation (Bock et al., 2015). A common scenario in many invasions is that small founder population sizes will often lead to reduced genetic diversity, and invading populations experience large environmental perturbations, such as changes in habitat and environmental stress (Lawson Handley et al., 2011).

An invasion process is composed of four main stages (transport, colonization, establishment, and spread) that need to be overcome by a population (e.g., Beck et al., 2008; Ochocki & Miller, 2017; Richardson, Pyšek, & Carlton, 2011). By reaching the next stage, an alien species gains a new status (e.g., Beck et al., 2008; Blackburn et al., 2011; Theoharides & Dukes, 2007). However, by breaking specific barriers and continuing to the next stage of invasion, a population may incur genetic variability loss (Lawson Handley et al., 2011). Population processes during an invasion are highly dynamic (e.g., Crooks, 2005; Facon et al., 2006; Theoharides & Dukes, 2007). Any actions to control an invasion should be attuned to this varying dynamic. All authors agree that prevention and early detection of potentially invasive organisms are most effective and economic (e.g., Blackburn et al., 2011; Cacho, Spring, Pheloung, & Hester, 2006; Veitch & Clout, 2002). However, the detection of such small populations is difficult. Failure to detect these populations may lead to their growth. This may lead to a demographic explosion, making it more problematic, or even impossible, to control an invasion. The extremely harsh abiotic conditions in the Antarctic put particular pressure on alien organisms. Many alien plant propagules reach the region due to human‐mediated transport (e.g., Hughes, Convey, Maslen, & Smith, 2010; Lityńska‐Zając, Chwedorzewska, Olech, Korczak‐Abshire, & Augustyniuk‐Kram, 2012; Cuba‐Díaz, Troncoso, Cordero, Finot, & Rondanelli‐Reyes, 2013; for data on the broader Antarctic, see McGeoch, Shaw, Terauds, Lee, & Chown, 2015). However, only a few of these nonindigenous species can survive even a single vegetation season in the Antarctic, reaching the status of casual alien plant (Smith, 1996; Smith & Richardson, 2011). Only one alien species, *Poa pratensis* L., survived for over 60 years on the Antarctic Peninsula before it was eradicated. However, this species was not able to reproduce sexually (Pertierra et al., 2017).

**Figure 1:**
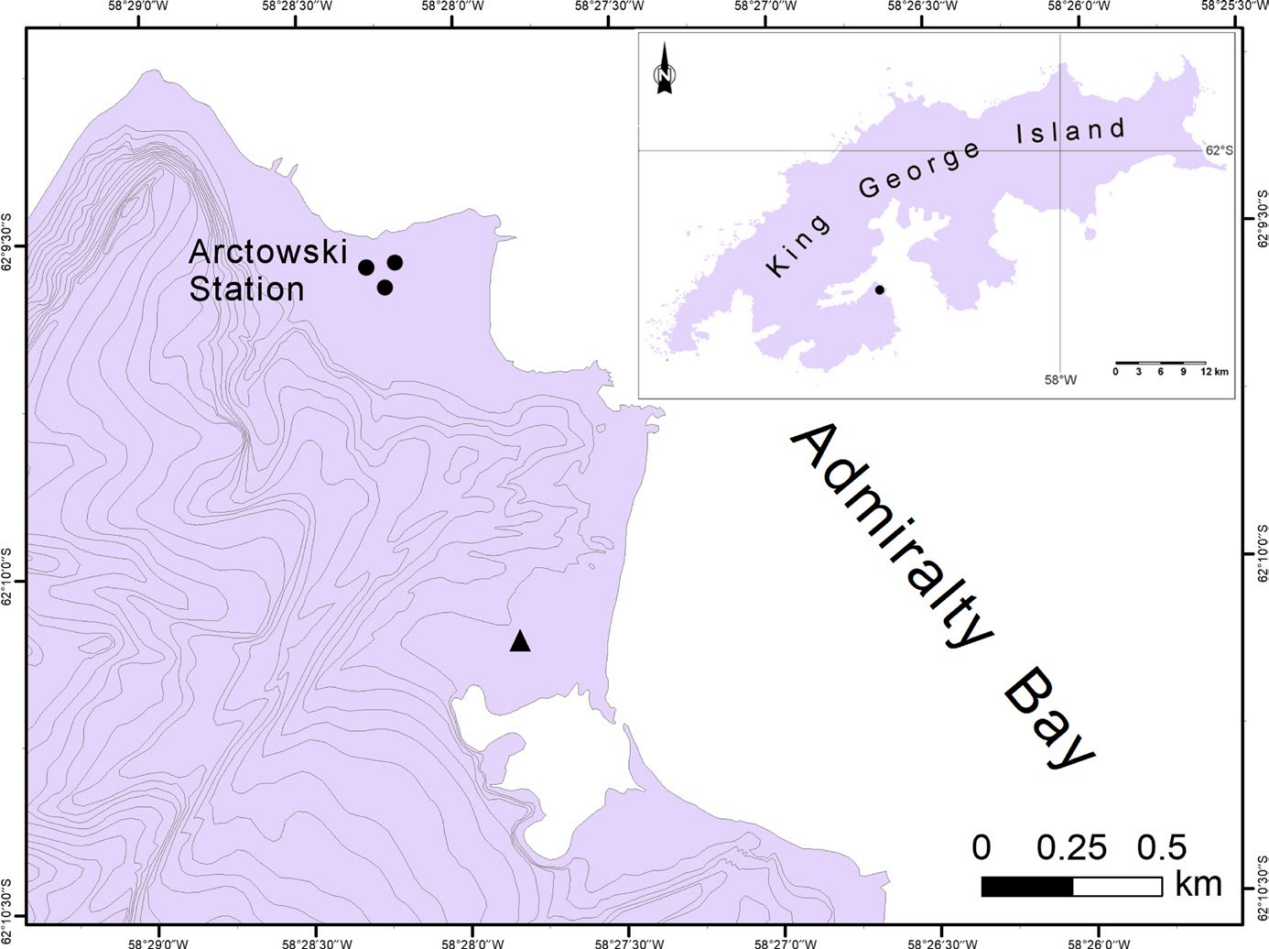
Location of *Poa annua* in the vicinity of Polish Antarctic Station Arctowski, ● Station, ▲ Forefield populations

Population demographic development has proven to be possible in the case of one nonindigenous plant species, *Poa annua* L. The species was recorded in several locations in the vicinity of the research stations along the Antarctic Peninsula (see Chwedorzewska et al., 2015; Molina‐Montenegro, Carrasco‐Urra, Acuña‐Rodríguez, Oses, & Chwedorzewska, 2014). The most numerous populations of the species have been observed since the 1985/6 austral summer at the Henryk Arctowski Polish Antarctic Station, King George Island, South Shetlands (Olech, 1996). The expansion of *P. annua* in the vicinity of “Arctowski” is well documented (Olech, 1996; Chwedorzewska 2008). In the austral summer of 2008/09, a population with numerous individuals of *P. annua* was recorded in a new location, 1.5 km from the “Arctowski” on the deglaciated moraines of the Ecology Glacier (Figure 1; Olech & Chwedorzewska, 2011). According to available historical data, one can make the hypothesis that at “Arctowski” the diaspores of *P. annua* originated from Poland, most likely from unsterilized soil for the greenhouse transported to the station in 1978 from the Botanical Garden in Warsaw‐Powsin. This is supported by observations conducted during 2000–2001 Polish Antarctic Expedition when emergence of *P. annua* seedlings was observed in the greenhouse building in a box containing soil destined for incineration (Chwedorzewska et al., 2015). A fundamental question arose, regarding the origin of this species, during our long‐term eradication program which started in 2014/2015 austral summer season (Galera, Chwedorzewska, & Wódkiewicz, 2017). Therefore, our first question was whether the new population on the deglaciated moraines of the Ecology Glacier originated from “Arctowski” population due to secondary dispersal, or whether it was a new introduction from a different source. Our second question was if the Ecology Glacier population proved to originate from the “Arctowski” population and how much of the species’ genetic variability had been transferred into the daughter population. The main goal of our study was to determine the level of genetic variation between the two Antarctic populations of *P. annua* and the Polish population which is the most probable source of primary introduction of this species at “Arctowski.”

## 2 METHODS

### 2.1 Sample collection

Samples of *P. annua* were collected during the austral summer season of 2008/9 from the population growing in the vicinity of the Polish Antarctic Station (62°09′34″S, 58°28′25″W; this locality is hereafter referred to as the *Station* population). Samples from the Polish population (from the Botanical Garden in Warsaw‐Powsin; 52°06′53″N, 21°05′65″E; hereafter referred to as *Warsaw*) were collected in May 2009. The population found growing on the Forefield of the Ecology Glacier within the Antarctic Specially Protected Area 128 (62°10′04″S, 58°27′49″W; hereafter referred to as *Forefield*; Figure 1) was sampled during the 2009/10 austral summer season. Fresh shoots of 96 individuals from each locality were collected from the *Warsaw* and *Station* populations. Due to the small size of *Forefield* population, only 30 individuals were analyzed. All samples were desiccated with silica gel and frozen at −70°C until DNA extraction.

### 2.2 DNA extraction and AFLP assay

Total DNA was extracted with the MagAttract^®^ 96 DNA Plant kit (Qiagen) following the manufacturer's recommendations. To assess genetic variability, we used the amplified fragment length polymorphism (AFLP) procedure (Vos et al., 1995) with modifications (Chwedorzewska, Bednarek, Puchalski, & Krajewski, 2002), using *Kpn*I/*Mse*I enzymes for the digestion of 500 ng of genomic DNA. After digestion, ligation of the appropriate adaptors was performed, followed by preselective and selective amplification steps. The selective amplification was carried out in the presence of 5′‐ (^32^P)‐labeled primers. Eight selective primer pair combinations were used (Table 1). The PCR products were separated on 5% polyacrylamide gel and visualized by exposure to X‐ray films at −70°C overnight. Two independent repeats of selective amplification and polyacrylamide gel electrophoresis were performed. AFLPs are dominant markers. Each amplification product (band) represents the phenotype at a single biallelic locus. Reproducible, clearly distinguishable bands were scored manually (two times by two independent persons) across all samples as either present (1) or absent (0) and recorded in the form of a binary matrix.

**Table 1:** Number of bands generated with the selected primer pairs for each analyzed population

Primer pair code	Detected bands			Polymorphic bands		
	Warsaw	Station	Forefield	Warsaw	Station	Forefield
CpXpG‐AGC/M‐CCA	32	29	25	11	7	1
CpXpG‐GGC/M‐CAA	27	28	25	14	8	4
CpXpG‐AGA/M‐CCC	28	28	27	9	7	6
CpXpG‐AGG/M‐CAG	59	55	49	33	23	12
CpXpG‐TGC/M‐CGG	18	14	14	7	0	0
CpXpG‐ACC/M‐CCA	78	69	62	43	27	19
CpG‐GGT/M‐CCG	13	13	13	0	0	0
CpG‐AGG/M‐CAT	16	16	16	4	2	2
Total	270	252	238	121	74	44

### 2.3 Data analysis

GenAlEx 6.5 (Peakall & Smouse, 2006, 2012) was used to evaluate allele frequencies; number of bands shared among individuals with a frequency greater or equal to 5%; number of unique bands; Shannon's Information Index (I); and expected heterozygosity (*H*_e_) for each population from binary data assuming Hardy–Weinberg equilibrium (Nei 1973, Bensch & Ĺkesson, 2005), percentage of polymorphic bands (P%). This software was also used to perform AMOVA (Analysis of Molecular Variance) and to estimate the Ф_PT_ value with 1,023 permutations and 20,000 bootstraps to evaluate statistical significance. The Tajima's *D,* Fu's *F*_S_ neutrality tests, and the mismatch distribution and demographic processes affecting the populations were estimated with the Arlequin software, version 3.11 (Excoffier, 2005; Fu 1997).

**Figure 2:**
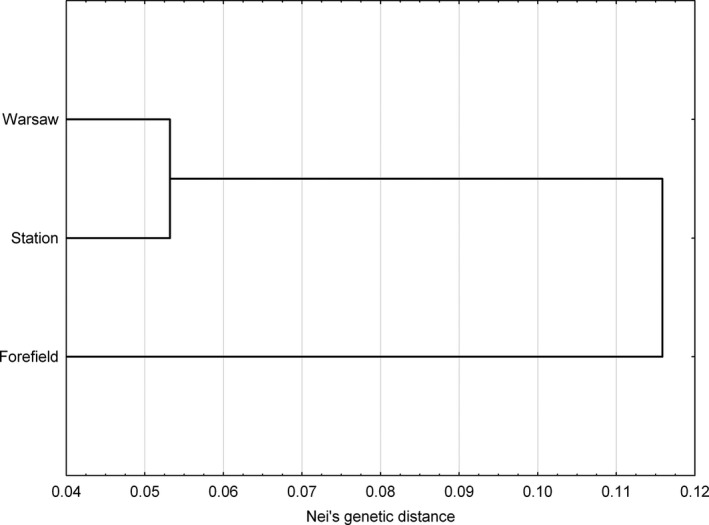
Unweighted pair‐group method with arithmetical averages dendrogram based on all amplified fragment length polymorphism products for all analyzed *Poa annua* populations

The bottleneck hypothesis was tested using the Bottleneck software (Cornuet & Luikart, 1996). The population structure was analyzed with Structure Harvester ver. 0.6.94 (Earl & Vonholdt, 2012) software set to the default parameters (Falush, Stephens, & Pritchard, 2007; Foll & Gaggiotti, 2008). The admixture model with correlated allele frequencies between populations was applied without using a priori information on population origin. Lambda (λ), the parameter of the distribution of allelic frequencies, was set to 1. A pilot study with the length of the burn‐in and MCMC (Markov chain Monte Carlo) of 100,000–300,000 each was performed. Finally, 500,000 burn‐ins and 500,000 iterations with 10 runs were carried out on the bioportal server (www.bioportal.uio.no) to quantify the amount of variation of the likelihood for each *K*. The range of possible *K*s tested was 1–10. In order to determine the optimal number of clusters (*K*), an ad hoc statistic Δ*K* (Evanno, Regnaut, & Goudet, 2005) was used. Additionally, in order to investigate patterns of genetic subdivision of analyzed populations of *P. annua*, dendrogram using UPGMA (unweighted pair‐group method with arithmetical averages) was created (STATISTICA 12.0, StatSoft Polska; Figure 2).

## 3 RESULTS

### 3.1 DNA polymorphism

The DNA profiling of all plant samples allowed the identification of 270, 252, and 238 AFLPs generated by eight primer pair combinations, for the *Warsaw*,* Station,* and *Forefield* populations, respectively (Table 1). Most of the bands were present with a frequency higher than 5% within the given population (Table 2).

**Table 2:** Amplified fragment length polymorphism marker characteristics shared among individuals from analyzed populations

Population	Warsaw	Station	Forefield
No. of bands	271	252	238
No. of bands frequency ≥5%	256 (94%)	248 (98%)	237 (99.6%)
No. of unique bands	15	1	

The population from Poland amplified 15 unique bands, while both the Antarctic populations had only one common band, which was absent from the *Warsaw* population. The level of polymorphism was highest in the *Warsaw* population and lowest in the *Forefield*. Also, the *Warsaw* population was more heterozygous than *Station* and *Forefield*, while the *Forefield* shows the lowest heterozygosity. According to the Shannon's Information Index, the available markers were informative enough to proceed with further analysis (Table 3).

**Table 3:** Intrapopulation genetic variability of the nonredundant AFLP's for the Warsaw, Station, and Forefield populations

Population	*N*	*N*_a_Mean ± *SE*	*N*_e_Mean ± *SE*	*I*Mean ± *SE*	*H*_e_Mean ± *SE*	P%
Warsaw	96	1.386 ± 0.032	1.185 ± 0.019	0.172 ± 0.015	0.112 ± 0.010	41
Station	96	1.177 ± 0.034	1.149 ± 0.018	0.131 ± 0.014	0.087 ± 0.010	27
Forefield	30	1.011 ± 0.033	1.111 ± 0.017	0.088 ± 0.013	0.061 ± 0.009	15

*N*, number of samples; *N*_a_, number of different alleles; *N*_e_, number of effective alleles; *I*, Shannon's Information Index; *H*_e_, expected heterozygosity; P%, percentage of polymorphic alleles (5% criterion).

### 3.2 Genetic structure

**Figure 3:**
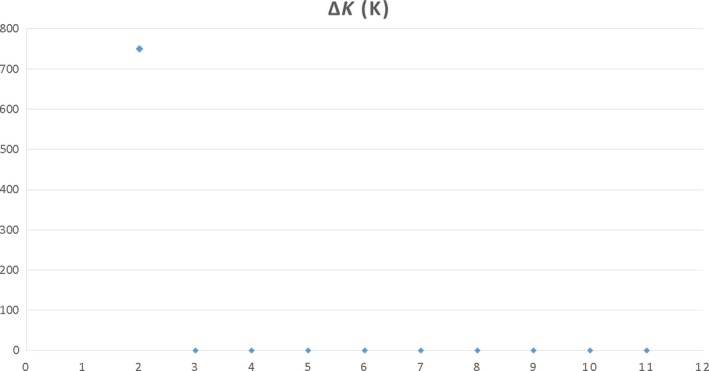
Estimated genetic structure for *K* = 2

Analysis of Molecular Variance revealed that all the populations differed from each other (Table 4). Nei's genetic distance and Ф_PT_ between the studied populations were biggest in the case of the *Forefield* and *Warsaw* populations and smallest between *Warsaw* and *Station* (Table 5). The evaluation of the agglomeration analysis based on the Δ(*K*) parameter revealed the presence of two groups of samples. No additional structuring was observed (Figure 3), what was also visible in the dendrogram (Figure 2).

**Table 4:** Partitioning of diversity found in *Poa annua* from all analyzed populations using AMOVA Φ_PT_ 0.376, *p *<* *.001 (9,999 permutations)

Source of variability	Sum of squares	Variance components	Percentage of variability
Among populations	1110.8	8.04	38
Within populations	2925.5	13.36	62
Total	4036.3	21.4	

**Table 5:** Nei's Genetic distance (GD), pairwise Ф_PT_, pairwise *F*_ST_ between analyzed populations

Population	Warsaw			Station		
	Nei's GD	Ф_PT_	*F*_ST_	Nei's GD	Ф_PT_	*F*_ST_
*Station*	0.053	0.283	0.283	–	–	–
*Forefield*	0.136	0.498	0.498	0.094	0.466	0.498

### 3.3 Neutrality tests and demography

Tajima's *D* and Fu's *F*_ST_ neutrality tests revealed that Tajima's *D* did not show any deviation from 0, while Fu's *F*_S_ was negative and significant for all populations (Table 6).

**Table 6:** Tajima's *D* test and Fu's *F*_S_ neutrality tests of characteristic for analyzed populations

Test	Description	Population			Statistics	
		Warsaw	Station	Forefield	Mean	*SD*
Tajima's *D* test	S	114	74	42	76.667	36.074
	Π	31.596	25.614	14.347	23.852	8.758
	Tajima's *D*	1.406	2.539	1.305	1.750	0.685
	Tajima's *D p* value	.890	1.000	.950	.946	.055
Fu's *F*_S_ test	Θ–π	31.596	25.614	14.347	23.852	8.758
	Expected no. of alleles	44.482	40.297	16.534	33.771	16.073
	*F*_S_	−23.958	−23.958	−19.470	−22.473	2.601
	*F*_S_ *p* value	.000	.000	.000	.000	.000

In the mismatch distribution test for demographic/spatial expansion, there were no significant SSD values, and all the samples had a very low raggedness index (Table 7).

**Table 7:** Mismatch analysis

Model	Statistics	Warsaw	Station	Forefield	Mean	*SD*
Demographic expansion	SSD	.0006	.0027	.0010	.0014	.0011
	Model (SSD) *p* value	.3270	.0230	.9110	.4203	.4513
	Raggedness index	.0013	.0023	.0049	.0028	.0018
	Raggedness *p* value	.6100	.1050	.8380	.5173	.3750
Spatial expansion	SSD	.0006	.0027	.0010	.0014	.0011
	Model (SSD) *p* value	.3050	.1000	.8860	.4003	.4457
	Raggedness index	.0013	.0023	.0049	.0028	.0018
	Raggedness *p* value	.6000	.1180	.8610	.5263	.3770

Arrangements of statistics for mismatch distribution and demographic/spatial expansion for all analyzed populations.

The three tests (Sing, Standardized, and Wilcoxon) for excess heterozygosity implemented in the bottleneck software produced significant *p* values based on the IAM model (Table 8).

**Table 8:** Testing bottleneck versus mutation drift equilibrium hypotheses for all analyzed populations

Population	Mutation model	SING Test	Standardized test	Wilcoxon test
Warsaw	IAM	Hee = 45.4	T2: 5.614	One tail of heterozygosity deficiency 1.0000
	Hd = 45	*p *=* *.0000	One tail of heterozygosity excess 0.0000
	He = 68		Two tail of homozygosity deficiency and excess 0.0000
Station	Hee = 29.87	T2: 8.134	One tail of heterozygosity deficiency 1.0000
	Hd = 14	*p *=* *.0000	One tail of heterozygosity excess 0.0000
	He = 60		Two tail of homozygosity deficiency and excess 0.0000
Forefield	Hee = 17.48	T2: 3.696	One tail of heterozygosity deficiency 0.99986
	Hd = 11	*p *=* *.00011	One tail of heterozygosity excess 0.00031
	He = 30		Two tail of homozygosity deficiency and excess 0.00361

Hee, Expected heterozygosity excess; Hd, Heterozygosity deficiency; He, Heterozygosity excess.

## 4 DISCUSSION

The *Kpn*I/*Mse*I platform was highly efficient in differentiating the analyzed populations as pointed out *I* value (Table 3). The Polish population exhibited 15 unique bands, highest level of polymorphism and heterozygosity in comparison with the *Station* and *Forefield* populations (Tables 2 and 3). According to available data (Chwedorzewska et al., 2015; Galera, Chwedorzewska, & Wódkiewicz, 2015; Galera et al., 2017; Olech, 1996; Olech & Chwedorzewska, 2011), one can hypothesize that the diaspores of *P. annua* probably came from Warsaw. But, Lityńska‐Zając et al. (2013) identified caryopses of *P. annua* among diaspores and phyto‐remains of 46 other plant species in cargo transported to Arctowski. Thus, it cannot be excluded that the Antarctic population was founded by multiple introductions from different sources, which is supported by the presence of one band exclusive to both Antarctic populations. Multiple introductions are a common feature of biological invasions (Dlugosch, Anderson, Braasch, Cang, & Gillette, 2015; Facon, Jarne, Pointier, & David, 2005). We suspect the same phenomenon in the case of the *Station* population (Lityńska‐Zając et al., 2012); therefore, intraspecific hybridization (i.e., an “admixture”) can play a role in the invasion success of *P. annua* in Antarctica. This process can change the distribution of phenotypes in a population, and the admixed individuals are able to outcompete their parental genotypes as a result of either heterosis effects, by creating new genotypes through recombination (Dlugosch et al., 2015; Facon et al., 2005), or via phenotypic plasticity (e.g., Lavergne & Molofsky, 2007).

The lowest variability of the *Forefield* population suggested genetic drift during the founding of the population. The demographic expansion was indicated by negative values of Fu's *F*s statistics and confirmed by an analysis of mismatch distribution, following an initial bottleneck or founder effect. Putative genetic drift affecting the new population may explain the observed data structuring. Together with the lack of unique bands for the *Forefield* population (in comparison with *Station*), this supports the hypothesis that the *Forefield* population was introduced directly from *Station* and did not originating as a new introduction. In order to study such effects using dominant markers, the infinite allele model can be used to test the mutation–drift versus the bottleneck hypothesis (Tero, Aspi, Siikamäki, Jäkäläniemi, & Tuomi, 2003). As expected, the *Forefield* population fulfilled the bottleneck hypothesis, or more likely the founder effect. However, it is difficult to perceive the difference between the bottleneck and founder effect with the application of dominant markers. In parallel to the demographic processes, the *Forefield* population may have been affected by some kind of selection processes. The most probable vector responsible for the establishment of the *Forefield* population is wind and/or human activity. This population is located in a place isolated by hills and at a substantial distance (approximately 1.5 km, Figure 1) from the *Station* population (Olech & Chwedorzewska, 2011); therefore, wind dispersal seems less probable, although it cannot be totally excluded. Consequently, seeds very likely were transferred on shoes or clothing of the personnel working at ASPA 128. This is also supported by our previous study showing that a great number of propagules were associated not only with cargo but also with personal clothes and field gear (Lityńska‐Zając et al., 2012). *Poa annua* flowers profusely (Galera et al., 2015) and produces numerous viable seeds under Antarctic conditions (Wódkiewicz, Galera, Giełwanowska, Chwedorzewska, & Olech, 2013; Wódkiewicz, Ziemiański, Kwiecień, Chwedorzewska, & Galera, 2014) which could be transported away from the *Station* population. A substantial number of *P. annua* tussocks were located at a heavily trampled area within Arctowski (Galera et al., 2017); thus, the soil containing small seeds may have been transferred on boots and transported to other areas. The low genetic diversity of the *Forefield* population suggests that the introduction was a single event. However, we cannot completely exclude multiple introductions followed by the limited establishment of transported seeds or the establishment of individuals specifically equipped with a narrow set of favored genes.

Comparisons of genetic variability of the only two Antarctic angiosperm *Deschampsia antarctica* Desv., Poacea (Chwedorzewska & Bednarek, 2011; Φ_PT_ = 0.031) and *Colobanthus quitensis* (Kunth) Bartl., Caryophyllaceae (Androsiuk, Chwedorzewska, Szandar, & Giełwanowska, 2015; *F*_ST_ = 0.164), with the local population of *P. annua* show that, the Antarctic populations of *P. annua* still have much higher levels of genetic variability, even in the *Forefield* population (Table 5). *Poa annua* in Antarctica is in the early stages of invasion, so the Antarctic populations are probably still unstable and undergoing dynamic demographic processes.

The polyploidy of *P. annua* may also inflate its intrapopulation genetic variability. This species is an allotetraploid and thought to be derived from a cross between *Poa infirma* H.B.K. and *Poa supina* Schrad., both 2*n* = 2*x* = 14 (Heide, 2001). Polyploids occur with greater frequency among invasive plants than among angiosperms in general (Brown & Marshall, 1981; Pandit, Tan, & Bisht, 2006; Prentis, Wilson, Dormontt, Richardson, & Lowe, 2008), with many allopolyploid hybrids among them (Lee, 2002). It is considered that polyploid hybrids tend to have greater fitness, possibly because of increased heterozygosity and reduced inbreeding depression (Soltis & Soltis, 2000), which can make them better colonizers than diploids, particularly under stress conditions (Prentis et al., 2008). This is supported by the high rate of allopolyploid species in the Arctic flora (Brochmann et al., 2004).

Our results show that the *Forefield* population was very likely introduced from the vicinity of Arctowski. Due to a limited number of individuals, this population was influenced by a bottleneck or founder effect and strong selection pressure, with parallel expansion. A critical factor in the success of this species is the ability to adapt rapidly to new environments following introduction (Galera et al., 2015; Wódkiewicz et al., 2014). The genetic variation is not necessary for an invasion to succeed (Dlugosch & Parker, 2008), because invasions can be followed by rapid adaptive evolution (e.g., Amsellem, Noyer, Le Bourgeois, & Hossaert‐McKey, 2000; Dlugosch & Parker, 2008). A particularly successful invasive population may originate from a former introduction by secondary dispersal (Lawson Handley et al., 2011).

Biological invasions have become regarded as “natural experiments,” offering unique insights into ecological and evolutionary processes occurring in real time (Lee, 2002; Sax et al., 2007). Understanding of these processes is crucial for implementing successful management policies. In a situation where much of the international scientific community's concern is devoted to minimizing the anthropogenic impact on Antarctic ecosystems (e.g., Hughes, Pertierra, Molina‐Montenegro, & Convey, 2015; McGeoch et al., 2015; Znój et al., 2017), the monitoring and eradication of even such a spatially limited invasion as in the case of *P. annua* on King George Island become an important conservation issue.

## CONFLICT OF INTEREST

None declared.

## AUTHOR CONTRIBUTIONS

KJC and MW conceived of this project. KJC collected plant samples and performed AFLP analysis. PTB and AP analyzed all data. MW, KJC, HG, and AZ wrote the manuscript. HG and AZ discussed ecological part of the manuscript. All authors commented on and contributed to the final manuscript.
